# Carbon ion radiotherapy of hepatocellular carcinoma provides excellent local control: The prospective phase I PROMETHEUS trial

**DOI:** 10.1016/j.jhepr.2024.101063

**Published:** 2024-03-11

**Authors:** Philipp Hoegen-Saßmannshausen, Patrick Naumann, Paula Hoffmeister-Wittmann, Semi Ben Harrabi, Katharina Seidensaal, Fabian Weykamp, Thomas Mielke, Malte Ellerbrock, Daniel Habermehl, Christoph Springfeld, Michael T. Dill, Thomas Longerich, Peter Schirmacher, Arianeb Mehrabi, De-Hua Chang, Juliane Hörner-Rieber, Oliver Jäkel, Thomas Haberer, Stephanie E. Combs, Jürgen Debus, Klaus Herfarth, Jakob Liermann

**Affiliations:** 1 Department of Radiation Oncology, Heidelberg University Hospital, Heidelberg, Germany; 2 Heidelberg Institute of Radiation Oncology (HIRO), Heidelberg, Germany; 3 National Center for Tumor Diseases (NCT), Heidelberg, Germany; 4 Clinical Cooperation Unit Radiation Oncology, German Cancer Research Center (DKFZ), Heidelberg, Germany; 5 Xcare Praxis für Strahlentherapie, Saarbrücken, Germany; 6 Department of Radiation Oncology, Heidelberg Ion Beam Therapy Center (HIT), Heidelberg University Hospital, Heidelberg, Germany; 7 Wilhelm-Conrad-Röntgen-Klinik Gießen, Universitätsklinikum Gießen und Marburg GmbH, Gießen, Germany; 8 Department of Medical Oncology, Heidelberg University Hospital, Heidelberg, Germany; 9 Liver Cancer Centre Heidelberg, Heidelberg, Germany; 10 Department of Gastroenterology, Infectious Diseases, Intoxication, Heidelberg University Hospital, Heidelberg, Germany; 11 Experimental Hepatology, Inflammation and Cancer Research Group, German Cancer Research Centre (DKFZ), Heidelberg, Germany; 12 Institute of Pathology, Heidelberg University Hospital, Heidelberg, Germany; 13 Department of General, Visceral & Transplantation Surgery, Heidelberg University Hospital, Heidelberg, Germany; 14 Department of Diagnostic and Interventional Radiology, Heidelberg University Hospital, Heidelberg, Germany; 15 Medical Physics in Radiation Oncology, German Cancer Research Center (DKFZ), Heidelberg, Germany; 16 Department of Radiation Oncology, Technical University of Munich (TUM), Munich, Germany; 17 Institute of Radiation Medicine (IRM), Helmholtz Zentrum München, Neuherberg, Germany; 18 German Cancer Consortium (DKTK), Partner Site Munich, Munich, Germany; 19 German Cancer Consortium (DKTK), Partner Site Heidelberg, Heidelberg, Germany

**Keywords:** SBRT, stereotactic ablative body radiotherapy, RILD, hadron therapy, HCC

## Abstract

**Background & Aims:**

Inoperable hepatocellular carcinoma (HCC) can be treated by stereotactic body radiotherapy. However, carbon ion radiotherapy (CIRT) is more effective for sparing non-tumorous liver. High linear energy transfer could promote therapy efficacy. Japanese and Chinese studies on hypofractionated CIRT have yielded excellent results. Because of different radiobiological models and the different etiological spectrum of HCC, applicability of these results to European cohorts and centers remains questionable. The aim of this prospective study was to assess safety and efficacy and to determine the optimal dose of CIRT with active raster scanning based on the local effect model (LEM) I.

**Methods:**

CIRT was performed every other day in four fractions with relative biological effectiveness (RBE)-weighted fraction doses of 8.1–10.5 Gy (total doses 32.4–42.0 Gy [RBE]). Dose escalation was performed in five dose levels with at least three patients each. The primary endpoint was acute toxicity after 4 weeks.

**Results:**

Twenty patients received CIRT (median age 74.7 years, n = 16 with liver cirrhosis, Child-Pugh scores [CP] A5 [n = 10], A6 [n = 4], B8 [n = 1], and B9 [n = 1]). Median follow up was 23 months. No dose-limiting toxicities and no toxicities exceeding grade II occurred, except one grade III gamma-glutamyltransferase elevation 12 months after CIRT, synchronous to out-of-field hepatic progression. During 12 months after CIRT, no CP elevation occurred. The highest dose level could be applied safely. No local recurrence developed during follow up. The objective response rate was 80%. Median overall survival was 30.8 months (1/2/3 years: 75%/64%/22%). Median progression-free survival was 20.9 months (1/2/3 years: 59%/43%/43%). Intrahepatic progression outside of the CIRT target volume was the most frequent pattern of progression.

**Conclusions:**

CIRT of HCC yields excellent local control without dose-limiting toxicity.

**Impact and implications:**

To date, safety and efficacy of carbon ion radiotherapy for hepatocellular carcinoma have only been evaluated prospectively in Japanese and Chinese studies. The optimal dose and fractionation when using the local effect model for radiotherapy planning are unknown. The results are of particular interest for European and American particle therapy centers, but also of relevance for all specialists involved in the treatment and care of patients with hepatocellular carcinoma, as we present the first prospective data on carbon ion radiotherapy in hepatocellular carcinoma outside of Asia. The excellent local control should encourage further use of carbon ion radiotherapy for hepatocellular carcinoma and design of randomized controlled trials.

**Clinical Trials Registration:**

The study is registered at ClinicalTrials.gov (NCT01167374).

## Highlights

• High-dose CIRT can safely spare non-tumorous liver without relevant toxicity.
• CIRT for HCC yields excellent local control (100% in this study).
• The promising results of Japanese and Chinese trials can be transferred to European centers and cohorts.
• Intrahepatic progression outside of the CIRT target volume remains the main challenge.

## Graphical abstract

**Figure:**
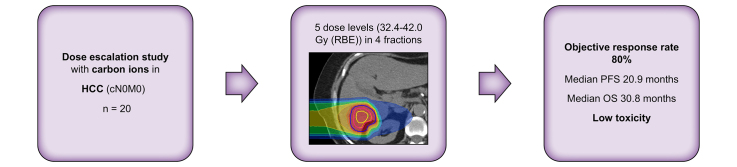

## Introduction

Globally, primary liver cancer (∼90% of which are hepatocellular carcinoma, [HCC]) has the fifth highest incidence of all cancers.^1^^,^^2^ HCC is endemic in some countries in East Asia and Africa. Incidence rates in Europe range from 2.7 to 10.5 per 100,000.^2^

Various local therapies such as radiofrequency ablation (RFA),^3^ microwave ablation (MWA), transarterial chemoembolization (TACE)^4^ and stereotactic body radiotherapy (SBRT)[5], [6], [7] have been established as treatment for unresectable HCC or as a bridge to transplantation. For the latter, RFA, TACE, and SBRT have shown comparable overall survival (OS) rates.^8^ Only few randomized controlled trials comparing radiotherapy (RT) to other local treatment modalities have been published. Proton beam therapy has been demonstrated as non-inferior to RFA in recurrent HCC^9^ and has shown superior local control (LC) and progression-free survival (PFS) compared with TACE.^10^ SBRT showed a trend towards better PFS and LC compared with TACE.^11^ In retrospective studies, LC after SBRT was comparable to RFA^12^ and superior to TACE.^13^ Although techniques such as RFA and MWA are only applied to lesions up to 5 cm,^3^^,^^4^^,^^14^ the feasibility and safety of SBRT have been demonstrated for median lesion diameters of 7–8 cm.^15^ Furthermore, vessel proximity or venous thrombosis play less of a role for SBRT eligibility.^2^^,^^12^^,^^16^

HCC mainly occurs in pre-damaged, cirrhotic, steatotic, or infected livers. Thus, sparing of non-tumor liver tissue is important to preserve liver function. The ratio of non-irradiated liver volume (defined as liver volume exposed to less than 1 Gy) to standard liver volume has been identified as an independent predictor of radiation-induced liver disease (RILD).^17^

Proton beam therapy and carbon ion radiotherapy (CIRT) have the potential to reduce toxicity and improve organ at risk (OAR) sparing over photon RT, as demonstrated recently in a systematic review.^18^ Ions omit the low dose bath characteristic for photon therapy. Radiation dose is usually low where beams enter the body and cumulates in a very localized maximum, the Bragg peak. Carbon ions offer a lower entry and integral dose for a given Bragg peak and much less lateral scattering compared with protons. This could help to further spare liver tissue. Additionally, because of their high linear energy transfer, carbon ions also have the potential to be more tumoricidal than photon and proton treatments, although this hypothesis is not confirmed for HCC by clinical data so far.^19^

CIRT planning is based on modeling of relative biological effectiveness (RBE). Three different biological models have commonly been used: the mixed beam model for passive scattering delivery,^20^ the local effect model (LEM)^21^ and the modified microdosimetric kinetic model (mMKM).^22^ The models differ in their approaches. The mixed beam model is based on *in vitro* cell survival curves, whereas LEM and mMKM are theoretical physical approaches.^23^

Although most publications on CIRT for HCC originate from Japanese centers, where the mixed beam model and more recently the mMKM have been used, carbon ion centers in Europe mostly rely on LEM I.^23^ Because of the different RBE models used in different ion centers and the lack of objective preclinical data as a basis for these models, prescription doses and single-center results from patients treated at one center cannot be generalized.^19^^,^^23^ Ion beams can be applied by passive scattering or by active scanning, which enables for more conformal dose distributions.^23^

To the best of our knowledge, only Japanese and Chinese centers have published prospective data on CIRT for HCC.^19^^,^^24^ It is therefore of utmost importance to evaluate CIRT of HCC at other, partly differently operating ion centers.

The aim of the present study was to evaluate safety and efficacy of CIRT for HCC at the Heidelberg Ion Beam Therapy Center (HIT) using the biological RBE-model LEM I and active raster scanning as the delivery method. The study was designed as a phase I dose escalation study with acute toxicity as the primary endpoint.

## Materials and methods

### Trial design

The PROMETHEUS trial (NCT01167374) was designed as a single-arm, single-center, phase I dose-finding study.^25^ The primary endpoint was toxicity 30 days after CIRT to identify the maximum tolerated dose. Patients were treated in four fractions. RBE-weighted fraction doses ranged from 8.1 to 10.5 Gy (RBE) using LEM I as the RBE-model. This unconventional prescription reflects the aimed 10–14 Gy (RBE) as calculated according to the approach of the Heavy-Ion Medical Accelerator (HIMAC) at the National Institute of Radiological Science, Chiba, Japan. This model is used in all Japanese heavy ion facilities and also in relevant studies.[26], [27], [28], [29] The RBE-models differ regarding the relationship between RBE-weighted and absorbed doses. Thus, the same prescription doses in the two models do not reflect similar absorbed doses. Consequently, the clinical outcomes of the same prescription dose in both models will likely differ. Steinsträter *et al.*^30^ have published factors to convert one dose into the other. In Table 1, prescription doses for both models and respective conversion factors are shown. Hereinafter, all doses mentioned are LEM I-based.

**Table 1:** Prescribed relative biological effectiveness (RBE)-weighted doses according to HIMAC and LEM I.

Dose according to HIMAC [Gy (RBE)] Fraction/total	Dose according to LEM I as used in the present study [Gy (RBE)] Fraction/total	Conversion factor
10.0/40.0	8.1/32.4	0.81
11.0/44.0	8.8/35.2	0.80
12.0/48.0	9.5/38.0	0.79
13.0/52.0	10.0/40.0	0.77
14.0/56.0	10.5/42.0	0.75

Modified from Steinsträter 2012.^30^

HIMAC, heavy-ion medical accelerator facility, National Institute of Radiological Science, Japan; LEM, local effect model; RBE, relative biological effectiveness.

At least three patients had to be treated at each dose level. Unless any dose-limiting toxicity (DLT) occurred, recruitment to the next higher dose level began. In the case of DLT, three more patients had to be recruited to the same dose level. DLT was defined as any irreversible grade IV toxicity within 30 days after study treatment. The study was approved by the local ethics committee (S-019/2010) and the German Federal Office for Radiation Protection (Bundesamt für Strahlenschutz). Informed consent was obtained from all study participants.

### Inclusion criteria

Patients fulfilling all the following criteria were considered for study recruitment: HCC confirmed histologically or diagnosed according to American Association for the Study of Liver Diseases guidelines^31^; absence of extrahepatic metastases (cN0M0); minimal distance of tumor edge to the intestines ≥1 cm; Karnofsky performance status scale (KPS) ≥60%. Patients with previous radiotherapy to the hepatobiliary system or prior malignancy other than HCC <2 years previously were excluded. Before study enrollment, each individual case was discussed by a multidisciplinary team including board-certified surgeons, gastroenterologists, medical oncologists, pathologists, interventional radiologists, and radiation oncologists. If a curative therapy was deemed feasible, patients were allocated to said therapy without exception according to international guidelines. Only patients that could not undergo surgery or ablation or declined these options were considered for study treatment.

Systemic therapy after CIRT was not part of the study protocol. It was neither mandatory, nor forbidden. As we considered SBRT to be a local ablative therapy, no patient received systemic treatment simultaneously to or immediately after study treatment.

### CIRT planning and application

Patients were immobilized in the supine position with vacuum pillows, immobilization of the arms, and abdominal compression. Both contrast-enhanced, multiphase computed tomography (CT) (slice thickness 3 mm) including native 4D-CT with eight phases and contrast-enhanced magnetic resonance imaging (MRI) were performed. Gross tumor volume (GTV) was delineated respecting all available imaging. A margin of 5 mm was added for the clinical target volume (CTV). An internal target volume (ITV) was created based on 4D-CT. Finally, a margin of 5–7 mm was added to obtain the planning target volume (PTV). In most of the patients, PTV margins were 5 mm isotropically except for 7 mm in the beam direction to account for range uncertainties.

OAR constraints were not explicitly defined in the study protocol. To avoid OAR overdosage, constraints were chosen according to international recommendations, for example by Emami *et al.*^32^ or the QUANTEC initiative.^33^

SyngoRT treatment planning software (Siemens, Erlangen, Germany) included biologic plan optimization. All treatment plans used one single beam. Irradiation was performed every other day. Patient positioning before therapy was verified using X-rays and CT imaging. CTs were performed either on a CT connected to the treatment room via a shuttle system^34^ or via in-room CT. Gating was applied if craniocaudal target volume motion exceeded 1.0–1.5 cm using a respiratory gating system (Anzai, Tokyo, Japan) to reduce interplay effects.[35], [36], [37]

### Follow up

Follow-up visits were scheduled every 4 weeks after CIRT for the first 3 months and then in intervals of 2–3 months. Follow-up imaging was performed with contrast-enhanced MRI. Blood samples were collected at every follow-up visit, if possible. Relevant parameters that were assessed included levels of albumin, bilirubin, international normalized ratio (INR), aspartate transaminase (ASAT), alanine transaminase (ALAT), gamma-glutamyltransferase (GGT), alkaline phosphatase (AP), cholinesterase (CHE), and alpha-fetoprotein (AFP).

### Outcome assessment

Toxicity was assessed and graded according to the Common Terminology Criteria for Adverse Events (CTCAE) 5.0. Radiological outcome was graded according to RECIST 1.1.^38^ Modified albumin–bilirubin (mALBI) grades were calculated to exclude the subjective parameters of Child-Pugh scores (CP).^39^^,^^40^

OS was defined as time from the start of radiotherapy until reported death of any cause. LC was defined as time from the start of radiotherapy until local failure. Local failure was defined as progression of target lesions according to RECIST 1.1 or new HCC lesions originating within the initial PTV.

PFS was defined as time from the start of radiotherapy until tumor progression of any kind. Intrahepatic progression was defined as any new or progressive HCC lesion outside of the initial PTV. LC and PFS were censored at the date of death. For patients lost to follow up, data were censored at the last follow-up visit (if a date of death could later be obtained from relatives or authorities, only LC and PFS were censored at the last follow up).

### Statistics

Statistical analysis was performed using SPSS version 27 (IBM Corporation, Armonk, NY, USA).

OS, LC, and PFS were analyzed by non-parametric Kaplan–Meier estimates. Univariate analyses were performed using Cox regression. Wilcoxon signed-rank test was used for pairwise comparison of dependent, ordinal or continuous, not normally distributed data. Bonferroni correction for multiple testing was applied. Values of *p* <0.05 were considered statistically significant.

## Results

### Patient and treatment characteristics

Between August 2011 and November 2020, 23 patients were enrolled in the study. Three patients dropped out before initiation of the study treatment (two withdrew their consent and one presented with nodal metastases). Twenty patients received the study treatment without any interruptions. In 2022, the study was closed in regard of a fully recruited final dose level and slow recruitment. Follow-up data were censored in December 2022.

Patient characteristics are summarized in Table 2. Notably, four patients had no cirrhosis. Alcohol was the most frequent cause of cirrhosis, whereas chronic viral hepatitis only accounted for 25%. Barcelona Clinic Liver Cancer (BCLC) stage C (n = 11, 55%) was most frequent because of reduced tumor-related performance score. Five patients had multiple HCC lesions. In three patients, these were located next to each other and could be covered by one PTV. In two patients, only one lesion was treated by CIRT whereas the other lesions were planned to be treated with TACE sequentially. Individual GTV and PTV mean and median doses are displayed in the Supplementary material. The median target volume fraction covered by at least 95% of the prescribed dose (V95%) was 100.0% for the GTV (range 99.6–100.0%) and 94.7% for the PTV (range 81.3–99.9%).

**Table 2:** Patient characteristics.

	n(%)
Patients	20 (100%)
Age, years. Median (range)	74.7 (55.7–83.6)
Karnofsky performance status at baseline	
100	4 (20%)
90	5 (25%)
80	7 (35%)
70	3 (15%)
60	1 (5%)
ECOG at baseline	
0	9 (45%)
1	10 (50%)
2	1 (5%)
Sex	
Female	5 (25%)
Male	15 (75%)
Cirrhosis	
Yes	16 (80%)
No	4 (20%)
Origin of cirrhosis	
Alcoholic	8 (40%)
Hepatitis B	1 (5%)
Hepatitis C	4 (20%)
Nutritional	1 (5%)
Cryptogenic	2 (10%)
Child-Pugh score at baseline (points/class)	
N/A (no cirrhosis)	4 (20%)
5/A	10 (50%)
6/A	4 (20%)
8/B	1 (5%)
9/B	1 (5%)
BCLC stage	
A1	1 (5%)
A2	1 (5%)
A4	1 (5%)
B	6 (30%)
C	11 (55%)
mALBI at baseline	
1	13 (65%)
2a	5 (25%)
2b	1 (5%)
3	1 (5%)
Histology	
Yes	11 (55%)
No	9 (45%)
AFP elevation (>8 IU/ml)	
Yes	4 (20%)
No	16 (80%)
Diagnosis of HCC based on:	
Histology	11 (55%)
Imaging and AFP elevation	3 (15%)
Two independent imaging methods	6 (30%)
Macrovascular invasion present in MRI	
Yes	1 (5%)
No	19 (95%)
Previous therapies (multiple, if applicable)	
None	9 (45%)
Surgery	7 (35%)
RFA	3 (15%)
MWA	1 (5%)
TACE	4 (20%)
Percutaneous ethanol injection (PEI)	1 (5%)
Sorafenib	1 (5%)
Dose levels (fraction/total) [Gy (RBE)]	
8.1/32.4	3 (15%)
8.8/35.2	3 (15%)
9.5/38.0	4 (20%)
10.0/40.0	3 (15%)
10.5/42.0	7 (35%)
HCC lesions in total	
1	15 (75%)
2	2 (10%)
≥3	3 (15%)
CIRT target lesions	
1	17 (85%)
2	1 (5%)
3	2 (10%)
GTV localization (liver segments)	
S IV	1 (5%)
S VI	6 (30%)
S VIII	4 (20%)
S II/III	2 (10%)
S V/VI	1 (5%)
S V/VIII	2 (10%)
S VI/VII	2 (10%)
S VII/VIII	1 (5%)
S IV/V/VIII	1 (5%)
Target volumes (accumulative). Median (range)	
GTV diameter (mm)	28 (12–65)
GTV volume (cc)	18.8 (2.0–125.7)
CTV volume (cc)	41.0 (9.1–191.6)
ITV volume (cc)	73.3 (11.3–275.8)
PTV volume (cc)	136.0 (33.1–437.5)
Whole liver volume (cc)	1,572.9 (830.3–2,815.7)
Liver mean dose (Gy)	6.7 (1.4–15.2)
Gated treatment	
Yes	7 (35%)
No	13 (65%)

AFP, alpha-fetoprotein; BCLC, Barcelona Clinic Liver Cancer; CIRT, carbon ion radiotherapy; CTV, clinical target volume; ECOG, Eastern Cooperative Oncology Group performance status; GTV, gross tumor volume; HCC, hepatocellular carcinoma; ITV, internal target volume; mALBI, modified albumin–bilirubin; MRI, magnetic resonance imaging; MWA, microwave ablation; PTV, planning target volume; RFA, radiofrequency ablation; TACE, transarterial chemoembolization.

### Toxicity

No DLT and only one toxicity exceeding grade II occurred. A patient of the lowest dose level had grade III GGT elevation at 12 months, synchronous with hepatic tumor progression. One patient of the second dose level presented with grade II AP elevation and ascites at the first follow up, likely as symptoms of asymptomatic RILD. At the next follow up, both ascites and AP elevation had spontaneously resolved. The most frequent adverse effects were fatigue and abdominal pain, shown in Table 3. Compared with baseline, CP, mALBI, albumin, bilirubin, INR, ALAT, ASAT, GGT, CHE, and AFP did not differ significantly at the last day of RT and at 1, 3, 6, and 12 months after CIRT (*p* >0.05, respectively). The only parameter varying significantly was AP at 12 months after CIRT (*p* = 0.035, baseline AP: median 92.5 U/L [range 63.0–298.0 U/L], AP at 12 months: median 114.0 U/L [range 74.0–201.0 U/L]).

**Table 3:** Toxicity.

	Baseline			During RT			1 month			2–3 months			>3 months (cumulated)		
Grade	I	II	III	I	II	III	I	II	III	I	II	III	I	II	III
Fatigue	3	0	0	6	2	0	7	0	0	8	0	0	10	1	0
Abdominal pain	1	0	0	1	0	0	2	0	0	1	0	0	4	1	0
Ascites	2	1	0	2	1	0	2	0	0	1	0	0	1	0	0
Nausea/vomiting	1	0	0	1	0	0	0	1	0	0	0	0	2	0	0
Diarrhea	0	0	0	0	0	0	1	0	0	1	0	0	1	0	0
Constipation	2	0	0	0	0	0	1	0	0	2	0	0	3	0	0
Erythema	0	0	0	2	0	0	1	0	0	2	1	0	1	0	0
Hyperpigmentation	0	0	0	0	0	0	0	0	0	0	0	0	1	0	0
Chest wall fibrosis	0	0	0	0	0	0	0	0	0	0	0	0	0	1	0
Weight loss	1	1	0	0	0	0	1	0	0	0	1	0	3	0	0
Anorexia	0	0	0	0	0	0	0	0	0	1	0	0	0	0	0
Hyperhidrosis	0	0	0	0	0	0	1	0	0	0	0	0	0	0	0
Xerostomia	0	0	0	0	0	0	1	0	0	1	0	0	0	0	0
Exertional dyspnea	0	0	0	0	0	0	0	0	0	0	0	0	2	0	0
INR elevation	3	0	0	1	0	0	0	0	0	1	0	0	2	0	0
Hypoalbuminemia	0	0	1	0	1	0	0	1	0	0	1	0	0	2	0
Bilirubin elevation	4	2	0	1	0	0	0	1	0	2	0	0	2	1	0
ASAT elevation	10	1	0	2	0	0	2	0	0	4	0	0	5	0	0
ALAT elevation	7	2	0	1	0	0	1	0	0	2	0	0	5	0	0
GGT elevation	5	4	9	1	0	0	2	0	0	3	0	0	3	0	1
AP elevation	8	2	0	0	0	0	0	1	0	1	0	0	5	0	0

Numbers given as absolute values (total patients: n = 20). Liver function parameters: baseline graded with regard to respective normal limits, follow up graded with regard to respective normal limits or baseline values (in the case of baseline exceeding normal limits) according to CTCAE 5.0.

ALAT, alanine transaminase; AP, alkaline phosphatase; ASAT, aspartate transaminase; CHE, cholinesterase; CTCAE, Common Terminology Criteria for Adverse Events; GGT, gamma-glutamyltransferase; INR, international normalized ratio.

One patient had an increase in CP before study treatment (from baseline, 6, to start of RT, 7). During 12 months of follow up, no CP increase above the level at start of RT was observed. In the same period, five patients had an increase in mALBI score of 1 grade each (two from 2a to 2b and three from 1 to 2a). In three of the five patients, mALBI decreased back to baseline levels within one or two follow-up visits. Six months after study treatment, one patient had a lower CP (5) than at baseline (6) and before the first fraction (7). At 12 months follow up, no patient had a CP lower or higher than at baseline. At both 6 and 12 months, no decline in mALBI score was observed. One patient had an increase of mALBI from 2a to 2b persistent at 6 and 12 months.

### Follow-up

Median follow up was 23.0 months for the overall cohort and 27.3 months for the survivors. At the time of analysis, two patients were still alive and two were lost to follow up. Four more patients were lost to clinical follow up, but their dates of death were certified by relatives or registry offices.

One patient died 5 days after the last fraction in a retirement home. According to the treating family doctor who also performed the postmortem examination, the patient had suffered from continuous decline of his general condition, dehydration, and hypotension over a longer period. The patient was 79.3 years old and multimorbid at baseline with a history of alcoholic cirrhosis (CP 9), esophageal varices grade III with status post upper gastrointestinal bleeding and variceal ligation, portal vein thrombosis, significant ascites, and pleural effusion as well as mild hepatic encephalopathy. After study treatment, he was treated with opioids by a palliative care unit at the retirement home without further diagnostic measures. The death was classified as most probably not related to the study treatment.

### Overall survival

Of the 16 patients that died, five died from tumor progression and four died from other, not HCC-related reasons. For seven patients, the reason of death could not be determined.

**Fig. 1:**
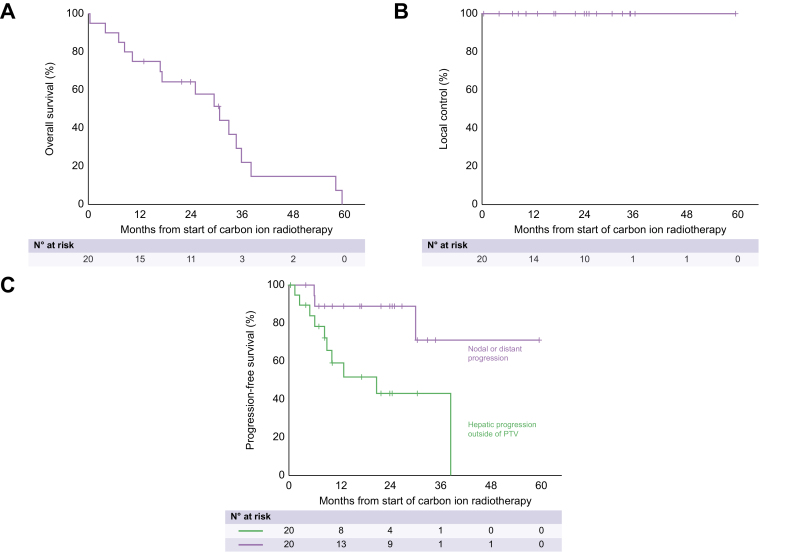
Kaplan-Meier plots of overall survival (A), local control (B) and progression-free survival (C) of the study cohort.

Median OS was 30.8 months (95% CI: 20.9–40.8 months). At 1/2/3/4 years, estimated OS was 75%/64%/22%/15% (Fig. 1A). Univariate analysis did not reveal any factor significantly associated with OS. A trend existed for CP (*p* = 0.073) and mALBI (*p* = 0.090).

### Local control

No local recurrence was observed (Fig. 1B). Two patients had new HCC lesions affecting the initial PTV during follow-up. In both patients, a new HCC nodule which developed clearly outside the PTV grew into the initial PTV volume.

**Fig. 2:**
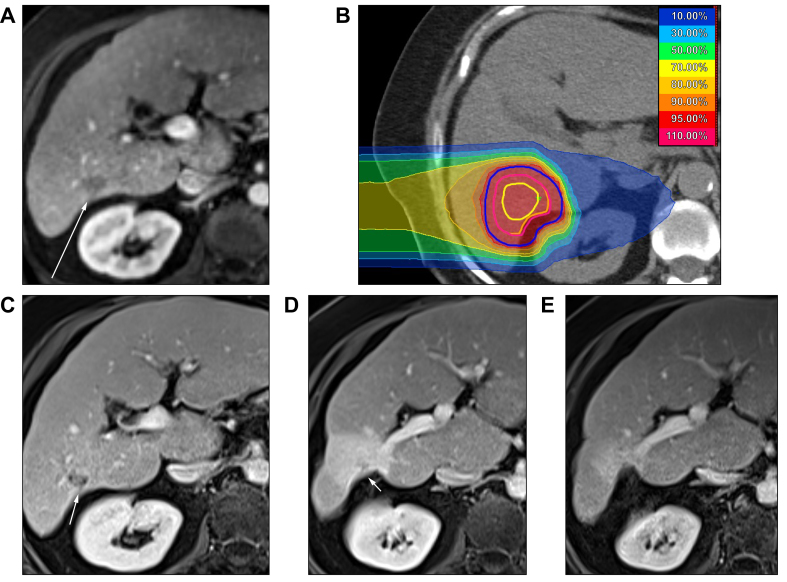
Carbon ion radiotherapy (CIRT) plan and MR images (T1, portal-venous contrast phase) at planning and during follow up. (A) Before CIRT; (B) CIRT plan with isodoses (prescribed dose 42.0 Gy [RBE] = 100.0%). Contours: yellow, GTV; orange, CTV; pink, ITV; blue, PTV. Note the conformal sparing of the adjacent right kidney. (C) One month after CIRT: stable disease. (D) Nine months after CIRT: partial remission and surrounding reaction with focal retraction of the liver capsule. (E) Two years after CIRT: complete remission and continuous retraction of the liver capsule, but decreasing liver tissue reaction. CTV, clinical target volume; GTV, gross tumor volume; ITV, internal target volume; PTV, planning target volume; RBE, relative biological effectiveness.

Five patients had complete remission and 11 patients had partial remission according to RECIST 1.1. Three patients had stable disease. One patient did not have any follow-up examinations because of early death. The objective response rate (ratio of patients with a complete or partial response) was 80%. Fig. 2 shows dose distribution and complete remission during follow up of a typical case.

### Progression-free survival

Ten patients showed hepatic progression, three of which also developed nodal or distant metastases. All patients with progressive disease had hepatic progression before or simultaneously to nodal or distant metastasis. Progression was treated by resection (n = 1), MWA (n = 1), CIRT (n = 1), TACE (n = 4), selective internal radiation therapy (SIRT, n = 1), sorafenib (n = 1), and best supportive care (n = 1).

The median PFS was 20.9 months (95% CI: 3.7–38.0 months, Fig. 1C). At 1/2/3 years, PFS was 59%/43%/43%. Univariate analysis did not reveal any factors significantly associated with PFS.

## Discussion

To the best of our knowledge, this is the first prospective trial using CIRT for HCC in Europe. In this dose-finding study, no DLT and practically no acute toxicities higher than CTCAE grade II occurred. The reported grade III GGT elevation 12 months after CIRT in a patient of the lowest dose level was most likely related to hepatic tumor progression outside of the irradiated volume. Half of the patient cohort developed hepatic progression with a median PFS of the total cohort of 20.9 months. As these patients required further therapies with potential hepatic, gastrointestinal and systemic toxicity, systematic analysis of late CIRT-specific toxicity was challenging. However, no additional toxicities clearly related to study treatment were reported later than 12 months. The final dose step was safely applied in all patients. One patient’s death 5 days after CIRT was classified as most likely not treatment-related. Previous studies also reported low, but occasional grade III toxicities such as hepatobiliary complications, radiation dermatitis, chest wall fibrosis, leukocytopenia, and gastric bleeding.^24^^,^[26], [27], [28], [29]^,^^41^

Liver function was not significantly impaired by CIRT, even in the light of high prevalence of cirrhosis. No CP elevation after CIRT occurred during the first year of follow up.

LC was excellent, without a single local recurrence. This is in line with the available data showing high LC rates of 92–100% at 1 year and 76.5–95.5% at 3 years for CIRT.^24^^,^[26], [27], [28], [29]^,^^41^ In two cohorts of comparable sizes with 21 and 23 patients, only one local recurrence each occurred.^24^^,^^28^

Based solely on the results of the present study, one might argue that the lowest dose level was the ideal one as no local recurrences occurred even in the lower dose levels. However, data on a clear dose–response relationship in photon SBRT of HCC remains inconclusive.^5^^,^^42^^,^^43^ Consequently, a benefit of higher, more ablative doses seems plausible. Additionally, Japanese Studies using CIRT in four fractions with total doses of 52.8–60.0 Gy (RBE) according to the HIMAC model (higher than the second highest and highest dose levels in the present work calculated with LEM I, see Table 1) still reported local recurrences. As the study treatment was well tolerated, the highest dose-level has been established as our in-house standard for CIRT in HCC.

In the present work, PFS and OS do not keep up with the excellent LC. One-year PFS was 59.1%. This reflects the status of HCC as a disease of the whole liver, at least in patients with cirrhosis.

The observed OS was significantly shorter compared with published data demonstrating OS rates of 90.3–95.4% at 1 year and 50.0–81.9% at 3 years.^24^^,^[26], [27], [28], [29]^,^^41^

Partially, this could be explained by more frail patients with lower performance status. Only 45% of patients presented with an Eastern Cooperative Oncology Group performance status (ECOG) 0 compared with 52–68% in other studies.^24^^,^^28^^,^^29^^,^^41^ Compared with one large retrospective cohort, BCLC stage was also worse in the present cohort with 15% A, 30% B, and 55% C compared with 63% A, 3% B, and 34% C.^41^ Regarding one combined analysis of two prospective trials with comparable BCLC stages, the difference in OS cannot be fully explained.^27^ Etiology of HCC generally differs significantly between Asian and American/European cohorts. This also applies to the present study with 40% of HCC related to chronic alcohol abuse and only 25% related to Hepatitis B or C, compared with 57–89% in Japanese and Chinese studies.^24^^,^[27], [28], [29]^,^^41^ Although etiology of HCC has not proven to be an independent prognostic factor so far,^44^ one could hypothesize that addict patients are generally more prone to a decline of their health condition and severe, potentially lethal comorbidities. In a large Danish cohort study with more than 19,000 alcohol-dependent patients and 180,000 control individuals, alcohol-dependent persons had increased hazards of all major somatic disease categories according to the International Classification of Diseases. Mortality hazard compared with the control group was 3.6 for females and 2.9 for males.^45^ In the present study, only five of the 16 deaths (31%) were HCC-related, whereas the reason of death was unknown in seven and not HCC-related in four patients. In two prospective Japanese studies, deaths were related to HCC or liver failure in 74%^27^ and 69%.^29^ Altogether, the reason for lower PFS and OS compared with Japanese and Chinese studies cannot be determined with certainty, but association with HCC etiology cannot be ruled out, at least for OS which does not depend solely on HCC-related events.

In some countries, allocation of patients to particle therapy instead of photon therapy might depend on the socioeconomic status of the patient. Our particle therapy center, however, has contracts with most public and private German health insurance funds. Thus, costs of particle therapy are covered for almost all patients in Germany. Although a referral bias cannot be ruled out completely, it seems very unlikely.

As a phase I dose-finding study, the present cohort was rather small. Complicated recruitment could be a challenge for future phase II or III studies. Nevertheless, the present results should encourage offering CIRT to a wider range of HCC patients enabling larger trials with improved recruitment.

Another limitation of the study is that potential local failures could have been masked by the small cohort and by the early deaths of patients that otherwise might have developed local recurrence during follow up.

In CIRT, RBE calculation is the basis for dose calculation and treatment planning. The underlying biophysical models usually rely on preclinical data.^23^ As demonstrated in a prospective study comparing proton beam therapy and CIRT in prostate cancer, these preclinical data do not always adequately reflect actual human *in vivo* conditions.^46^ Wrong assumptions can lead to under- or overdosage in both tumors and OAR. The validity of proposed conversion factors^30^ should thus be clinically evaluated. Given the excellent local control and the low toxicity, the established conversion factors seem to be valid.

Japanese carbon ion centers have also switched to active scanning techniques recently.^23^^,^^47^ The clinical data presented here with only few mild increases in Child-Pugh and mALBI scores demonstrate adequate sparing of surrounding liver tissue by active scanning beam delivery, although a direct comparison of both techniques was not part of this work.

## Conclusions

CIRT of HCC provides excellent local control with only mild, not dose-limiting toxicities. Because of the physical and biological effects of CIRT, healthy liver tissue could effectively be spared. The promising results of this dose-finding study should encourage larger randomized trials to compare CIRT with established local ablative therapies.

## Financial support

The authors have not received any financial support for the present study.

## Authors’ contributions

Development of the study: DH, SEC, KH, JD. Treatment of patients: PHS, PN, SBH, KS, FW, TM, ME, DH, CS, MTD, TL, PS, AM, DC, JHR, OJ, TH, SEC, JD, KH, JL. Statistical analyses: PHS. Data curation and analysis: PHS, PN, JL. Writing of the manuscript: PHS, PN, PHW, KS, ME, CS, MTD, TL, PS, AM, OJ, KH, JL.

## Data availability statement

Clinical patient data cannot be shared openly. Insight into pseudonymized data can be gained on request from the corresponding author.

## Disclaimer

Parts of this work have been submitted to the yearly conference of the German Society of Radiation Oncology (Deutsche Gesellschaft für Radioonkologie, DEGRO). The abstract has been accepted and will be presented as an oral presentation on June 22, 2023 in Kassel, Germany.

## Conflicts of interest

PH and JL are funded by the Physician-Scientist Program of Heidelberg University, Faculty of Medicine. PH received fees for an advisory board from NovoCure GmbH. FW received speaker fees from AstraZeneca and Merck Sharp & Dohme. JD received grants from Accuray International Sàrl, Merck Serono GmbH, CRI – The Clinical Research Institute GmbH, View Ray Inc., Accuray Incorporated, RaySearch Laboratories AB, Vision RT limited, Astellas Pharma GmbH, Astra Zeneca GmbH, Solution Akademie GmbH, Ergomed PLC Surrey Research Park, Siemens Healthcare GmbH, Quintiles GmbH, NovoCure, Pharmaceutical Research Associates GmbH, Boehringer Ingelheim Pharma GmbH Co, PTW-Freiburg Dr Pychlau GmbH, Nanobiotix A.A. and IntraOP Medical outside the submitted work. JHR received speaker fees and travel reimbursement from ViewRay Inc., travel reimbursement from IntraOP Medical and Elekta Instrument AB, and a grant from IntraOP Medical outside the submitted work.

Please refer to the accompanying ICMJE disclosure forms for further details.
