# Supplementary material for: Carbon ion radiotherapy of hepatocellular carcinoma provides excellent local control: The prospective phase I PROMETHEUS trial
Source: JHEP Rep. 2024 Mar 11;6(6):101063. doi: 10.1016/j.jhepr.2024.101063 (PMC11087711; doi:10.1016/j.jhepr.2024.101063)
Supplement: Multimedia component 1 [file mmc1.pdf]

Carbon ion radiotherapy of hepatocellular carcinoma provides excellent local control: The prospective phase I PROMETHEUS trial

Philipp Hoegen-Saßmannshausen, Patrick Naumann, Paula Hoffmeister-Wittmann, Semi Ben Harrabi, Katharina Seidensaal, Fabian Weykamp, Thomas Mielke, Malte Ellerbrock, Daniel Habermehl, Christoph Springfeld, Michael T. Dill, Thomas Longerich, Peter Schirmacher, Arianeb Mehrabi, De-Hua Chang, Juliane Hörner-Rieber, Oliver Jäkel, Thomas Haberer, Stephanie E. Combs, Jürgen Debus, Klaus Herfarth, Jakob Liermann

Table of contents

Table S1..2

Table S1: Mean and median doses for gross tumor volume (GTV) and planning target volume (PTV) in all patients

Patient Nr.	GTV mean dose	GTV median dose	PTV mean dose	PTV median dose
1	32.4	32.4	32.3	32.4
2	32.4	32.4	32.2	32.4
3	32.5	32.5	31.7	32.3
4	34.1	34.1	33.4	34.0
5	35.2	35.2	34.7	35.0
6	35.2	35.2	34.7	35.0
7	38.0	38.0	37.6	37.9
8	38.0	38.0	37.7	37.9
9	38.1	38.1	37.7	37.9
10	40.1	40.1	39.7	39.9
11	40.1	40.1	39.6	39.8
12	38.1	38.1	37.4	37.8
13	39.9	39.9	39.1	39.7
14	42.0	42.0	41.4	41.8
15	42.1	42.1	41.5	41.8
16	42.0	42.0	41.5	41.9
17	41.9	41.9	41.8	41.9
18	41.9	41.9	41.8	42.0
19	42.5	42.5	42.1	42.1
20	42.4	42.4	41.9	42.0
